# Modeling Temperature Effects on Population Density of the Dengue Mosquito *Aedes aegypti*

**DOI:** 10.3390/insects10110393

**Published:** 2019-11-07

**Authors:** Fadoua El Moustaid, Leah R. Johnson

**Affiliations:** 1 Department of Biological Sciences, Virginia Polytechnic Institute and State University, Blacksburg, VA 24060, USA; lrjohn@vt.edu; 2 Global Change Center, Virginia Polytechnic Institute and State University, Blacksburg, VA 24060, USA; 3 Department of Statistics, Virginia Polytechnic Institute and State University, Blacksburg, VA 24060, USA

**Keywords:** mathematical modeling, mosquito density, temperature, dengue, mosquito-borne disease

## Abstract

Mosquito density plays an important role in the spread of mosquito-borne diseases such as dengue and Zika. While it remains very challenging to estimate the density of mosquitoes, modelers have tried different methods to represent it in mathematical models. The goal of this paper is to investigate the various ways mosquito density has been quantified, as well as to propose a dynamical system model that includes the details of mosquito life stages leading to the adult population. We first discuss the mosquito traits involved in determining mosquito density, focusing on those that are temperature dependent. We evaluate different forms of models for mosquito densities based on these traits and explore their dynamics as temperature varies. Finally, we compare the predictions of the models to observations of *Aedes aegypti* abundances over time in Vitòria, Brazil. Our results indicate that the four models exhibit qualitatively and quantitatively different behaviors when forced by temperature, but that all seem reasonably consistent with observed abundance data.

## 1. Introduction

Mosquitoes transmit multiple pathogens, such as malaria, dengue, and Zika, that are responsible for significant death and morbidity in humans, making these animals among the most lethal to humans [1,2]. In particular, dengue is a life-threatening disease caused by dengue virus spread by *Aedes* species mosquitoes, including the yellow fever mosquito, *Aedes aegypti* [3,4]. Currently, there is not an effective vaccine or cure for dengue. Instead, dengue prevention relies solely on vector control and avoidance [5]. Thus, methods to improve our current prevention and control strategies are sought in order to reduce the severity of ongoing outbreaks and prevent them from occurring.

Mathematical models are important tools for understanding how both intrinsic factors (such as host susceptibility) and extrinsic factors (such as environmental conditions or interventions) impact the dynamics of infectious diseases, and of vector-borne diseases (VBDs) in particular [6]. Models for VBDs run the gamut from simple to complex, depending on the goals of the model [7]. Often, the primary modeling goal is to understand how the dynamics of mosquito-borne diseases respond to extrinsic factors such as climate/temperature or to explore the impacts of potential prevention and control strategies [8,9,10,11]. For example, Brand et al. [12] explored how more accurately predicting dengue transmission probability is valuable when deciding the appropriate control measures for a given outbreak. Similarly, predicting mosquito density and location is important when implementing an insecticide spraying strategy to prevent an outbreak [13,14]. These types of predictions require a deep understanding of the ecology of mosquitoes [15].

Although multiple mosquitoes could potentially transmit dengue, the primary vector globally is *Aedes aegypti*, with *Aedes albopictus* mostly a secondary vector (although it transmits widely in some Asian countries such as India) [16,17]. Mosquitoes go through three juvenile life stages (egg, larvae, and pupae) before they emerge as adults. The time spent within each juvenile life stage varies between species because of life history and genetic differences. There is also within-species variation as each of the life stages is sensitive to environmental conditions [4]. Because of this sensitivity to climate factors at each of their life stages, the dynamics of the mosquitoes, and thus of dengue transmission, is closely coupled to environmental variation. Thus, it is important to understand and include the role of environmental factors on mosquitoes when building models of dengue (or other mosquito-borne pathogens) transmission [17]. In particular, temperature is an important determinant of multiple mosquito life history traits, and so, it has a knock-on impact on mosquito density and on transmission.

In many models of mosquito-borne disease transmission, the dynamics of the mosquito population have been ignored (i.e., assumed to be constant) or treated very simply [7]. For example, a sine formula has been used to estimate mosquito density while taking into account possible seasonal fluctuations as a response to temperature changes [18,19]. In a review of the literature conducted by Legros et al. [20], they highlighted the importance of further investigating density dependence for *A. aegypti* and the need for further empirical studies to reduce our uncertainty when estimating mosquito density. Some progress has been made on this front. Although models for VBD transmission that include the dynamics of the density of vectors typically ignore the underlying characteristics of each stage, as well as the role of climate factors, in some models, approximations that depend on adult traits or a subset of larval traits have been used [9,11,21,22,23].

In this paper, we are interested exploring the extent to which including more details of mosquito life histories in a dynamical model impacts our predictions of female mosquito density, which should have knock-on effects for predictions of transmission and for the effects of intervention strategies aimed at controlling mosquitoes. To this end, we build a dynamical model of mosquito population dynamics that explicitly includes temperature-dependent traits for all life stages of the mosquitoes. We investigate the effect of temperature on mosquito life stages (based on published data) and incorporate these into our dynamical model. We then compare this model with three other models estimating mosquito abundance that rely on less (or no) trait data and explore their responses to temperature. We finally compare the predictions of all four models to data on weekly estimates of *A. aegypti* populations in Vitòria, Brazil. We discuss the advantages and disadvantages of the various modeling approaches in terms of both making predictions at a particular location and generalizing to other locations.

## 2. Methods

### 2.1. Dynamical Model of Mosquito Density Including Traits

**Figure 1:**
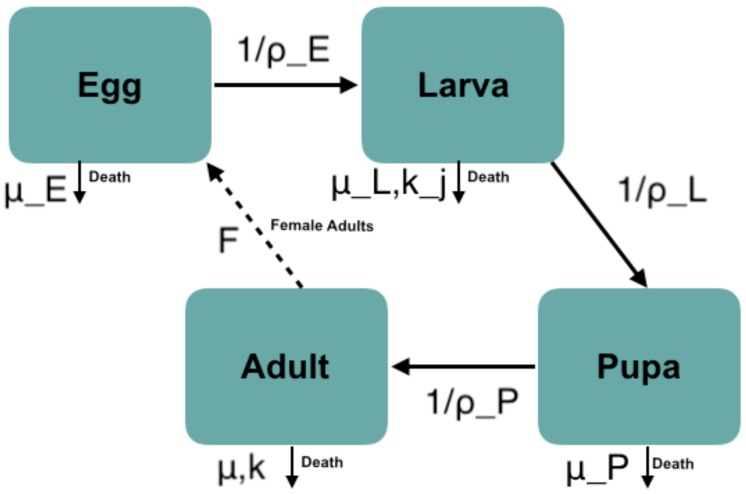
A graphical representation of the mosquito life cycle including eggs, larvae, pupae, and adults. Note that only female adults provide new eggs to the population, and the adult compartment represents just the female subclass. Arrows represent the movement between stages due to development, as well as the removal from the stage due to mortality.

We used a system of ordinary differential equations to describe mosquito vector dynamics at each of the four life stages, namely: eggs ($E_{g}$); larvae (*L*); pupae (*P*); and adults (*A*) (Figure 1). As only adult females lay eggs and take blood meals, most transmission models focus solely on the density of females mosquitoes. To be consistent in our comparisons later on, we retained this convention, and only modeled adult females, but we included both sexes in earlier stages.

Each day, every living adult female ($V_{d}\left(t\right)$) is assumed to produce a fixed number of eggs, *F*. A proportion of eggs ($E_{g}\left(t\right)$) dies at a temperature-dependent rate $\mu_{E}$, while a proportion develops into larvae (L(t)) at a temperature-dependent rate $1/\rho_{E}$ (Equation (1)). We assumed that larval death depends on two factors: over-crowding at a rate $k_{j}$; and temperature at a rate $\mu_{L}$. The surviving larval population moves to the pupal stage (P(t)) at a temperature-dependent rate $1/\rho_{L}$ (Equation (2)). Pupae either die at a temperature-dependent rate, $\mu_{P}$, or develop to the adult stage at a temperature-dependent rate, $1/\rho_{P}$ (Equation (3)). We further assume that 50% of the pupal population emerges as female adults. Female adults then experience mortality from a combination of temperature-dependent, μ, or a temperature-independent, *k*, causes (Equation (4)). Thus, our model equations are given by:

(1) $$\begin{array}{cc} \frac{dE_{g}}{dt} & =F\;A-\left(\mu_{E}+1/\rho_{E}\right)E_{g} \end{array}$$

(2) $$\begin{array}{cc} \frac{dL}{dt} & =E_{g}/\rho_{E}-\mu_{L}L\left(1+k_{j}L\right))-L/\rho_{L} \end{array}$$

(3) $$\begin{array}{cc} \frac{dP}{dt} & =L/\rho_{L}-\left(\mu_{P}+1/\rho_{P}\right)P \end{array}$$

(4) $$\begin{array}{cc} \frac{dV_{d}}{dt} & =0.5\times P/\rho_{P}-\left(\mu +k\right)A. \end{array}$$

The total female mosquito density is $V_{d}\left(t\right)$, our focus for subsequent analyses, which may be obtained via numerical integration of this system of equations. Note that the dynamics of $V_{d}\left(t\right)$ implicitly depends on the details of the other life history stages that precede it.

### 2.2. Alternative Models for Mosquito Density

**Figure 2:**
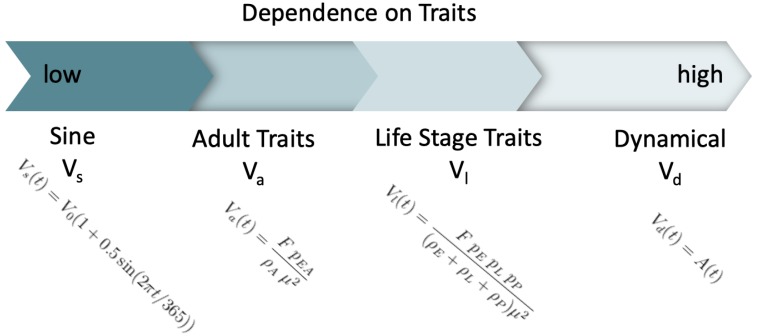
Traits’ dependence spectrum showing the evaluated density forms ranging from low to high traits’ dependence. First, the sine form $V_{s}$, second the adult traits density $V_{a}$, third the life stage traits’ density $V_{l}$, and the dynamical density $V_{d}$ last.

Our model is but one of many possible models that may be used to mathematically describe the dynamics of mosquito abundances. We were interested in comparing this proposed model with three other temperature-dependent mosquito density forms that rely on different amounts of biological detail and information. In Figure 2, we show the spectrum of the four densities we will compare. Our dynamical model is the most dependent on life history traits of the mosquitoes.

The model we considered second is an approximation that assumes a stationary and constant mosquito population, but still depends on the life stages traits [21]. We denote $V_{l}$ the density, given by:

(5) $$V_{l}\left(t\right)=\frac{F\;p_{E}\;p_{L}\;p_{P}}{\left(\rho_{E}+\rho_{L}+\rho_{P}\right)\mu^{2}}$$

where $p_{E}\;p_{L}\;p_{P}$ and $\rho_{E}+\rho_{L}+\rho_{P}$ are mortality rates and development times for eggs, larvae, and pupae, respectively. *F* and μ are defined as in the dynamical model above.

The third form we compared is an approximation of mosquito density that has been used in other vector-borne disease (VBD) models [9,11,24,25], where density is dependent on adult thermal traits:

(6) $$V_{a}\left(t\right)=\frac{F\;p_{EA}}{\rho_{A}\;\mu^{2}}$$

where $p_{EA}$ is the probability from eggs to adults and $\rho_{A}$ is adult mosquito development time.

Our final model is the most simple, in that it simply represents a seasonal pattern of the vector population, without explicitly including either temperature or traits. This form was used by Bacaër [18], Bacaër and Guernaoui [19] and represents the seasonally varying vector population as a simple sine function:

(7) $$V_{s}\left(t\right)=V_{0}\left(1+0.5\sin\left(2\pi t/365\right)\right)$$

where $V_{0}=mean\left(V\left(t\right)\right).$ We note that this density form is the same as the sine form used for temperature, which means that this form has the same temperature fluctuations and does not depend on temperature directly.

### 2.3. Parameterization and Numerical Analysis

We assumed that all the vector traits included in the model depend on temperature except the temperature-independent mortalities: *k* for adult mosquitoes and $k_{j}$ for larvae. The temperature dependence in traits is represented by unimodal thermal curves previously fit to trait data using a Bayesian method explained in detail in [24]. We used hump-shaped curves, a Brière, or a quadratic, to represent our temperature-dependent mosquito traits. The Brière curve is determined by the equation $\alpha T\left(T-T_{Min}\right)\sqrt{T_{Max}-T}$, the concave-down quadratic by the equation $qd\left(T-T_{0}\right)\left(T_{m}-T_{0}\right),$ and the concave-up by the equation $inter-slope\;T+qd\;T^{2}.$ These forms are symmetric (quadratic) and asymmetric (Brière) unimodal curves used by Mordecai et al. [24] to fit thermal curves to *A. aegypti* and *A. albopictus* adult mosquito traits using a Bayesian fitting method. Table 1 summarizes all the parameters used, their values, and references. These fitted curves describe each trait’s response to temperature from 0 °C to 50 °C. For the thermal curves’ function values, see Table 1, and for the plots of all curves, see Appendix A.

We assumed that across a year, all temperature-dependent parameters and variables are driven by the following temperature sine function capturing seasonality over a year:

(8) $$T\left(t\right)=T_{0}\left(1+B\sin\left(2\pi t/365\right)\right).$$

Here, $T_{0}$ is the starting temperature and *B* is the amplitude of the sine wave. For models $V_{l}\left(t\right)$ and $V_{a}\left(t\right)$. the density over time can be calculated simply by plugging in the temperature at each time based on this function. That is, we defined the temperature function, T(t), with *t* being days of the year. Then, we evaluated each parameter at each temperature using formulas from Table 1. $V_{s}\left(t\right)$ is evaluated directly.

Solving for the dynamics of $V_{d}\left(t\right)$ requires numerical integration of Equations (1)–(4) as they are being forced by the sinusoidal temperature function with each parameter defined at each temperature using formulas from Table 1. We used the MATLAB software ode solver, ode45, to solve the ode model numerically. First, we evaluated the model dynamics at one constant temperature $T_{0}$ until they reached equilibrium. We then used those steady state values as initial conditions for the temperature varying model.

Throughout our analysis, we tuned the temperature-independent parameters to obtain reasonable results. For the dynamical model, we adjusted *k* and $k_{j}$ to make the mean of $V_{d}$ close to the other densities. For the sine density $V_{s},$ we adjusted the amplitude and starting density. However, $V_{l}$ and $V_{a}$ depend solely on temperature-dependent thermal curves; thus, we did not adjust any of their parameters.

## 3. Results

We started our model analysis by exploring the dynamics of our full model (i.e., Equations (1)–(4)) under time varying temperatures. We then compared the predictions of the four models of total adult female mosquito densities.

### 3.1. Exploration of Dynamical Model Behavior

**Figure 3:**
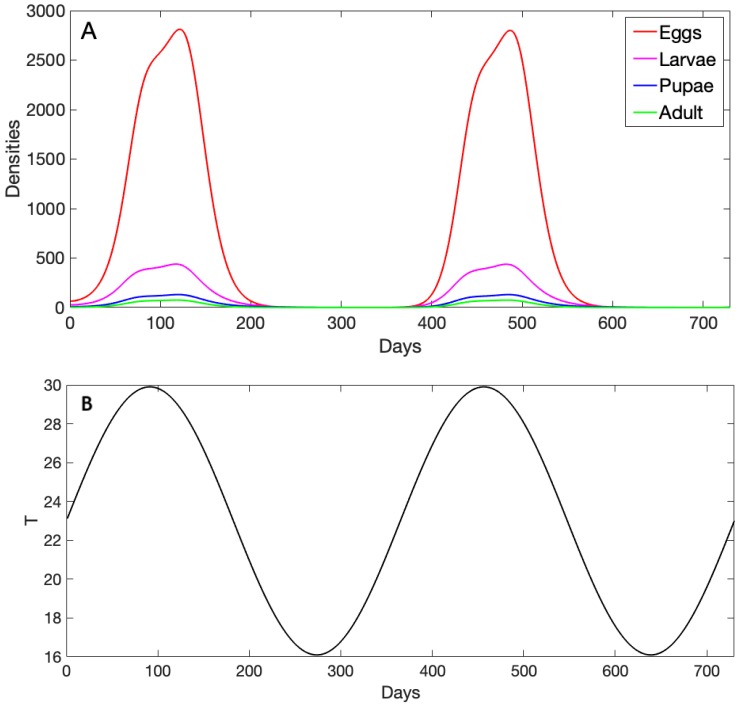
(**A**) Daily mosquito life stage densities evaluated for 730 days (two years). The lines show the numerical solution of our dynamical model for eggs, larvae, pupae, and female adult life stages, and the parameter values used here are given in Table 1. (**B**) The corresponding daily temperature given by a sine function ($T_{0}(1+B\sin(2\pi t/365$) with $T_{0}=23$ and B=0.3) evaluated for 730 days (two years) used to evaluate our thermal traits incorporated into the dynamical model.

We specified initial densities for each life stage and solved our model numerically as described in the previous section. We assumed that that the temperature curve had a mean temperature of 23 °C and ranged between 18.67 °C and 30 °C (Figure 3B), and it began on Day 0 at its mean (so that Day 0 was in the spring). Within this range, *A. aegypti* trait values kept the population from dying out completely. The juvenile population densities (eggs, larvae, and pupae; Figure 3A) initially increased exponentially as temperature increased to the 26–28 °C range (through ∼Day 75). As temperature approached its peak of 30 °C (∼Day 90), the growth rate was reduced across life stages before they resumed their increase as temperature began to lower again. As the temperature continued to drop (from ∼Day 120 onward in the year), densities decreased rapidly and reached their minimum. We show two years of dynamics to illustrate that although densities seemed to be zero, they did not in fact completely fade out. They only became very low, and as soon as temperature increased again, they grew, as we can see during the second-year phase.

### 3.2. Comparison of Mosquito Density Patterns across Models

Next, we compared the adult dynamics across all four models, again driven by sinusoidal temperature fluctuations. Recall the four densities, namely $V_{d}$ the adult female population result from our dynamical model, $V_{l}$ the life stage trait-dependent vector population, $V_{a}$ the adult trait-dependent vector population, and $V_{s}$ the sine wave approximation of the vector population. We chose two temperature regimes to illustrate the variety of patterns and predictions these models can exhibit. The results showed differences in both the values and patterns of these densities.

**Figure 4:**
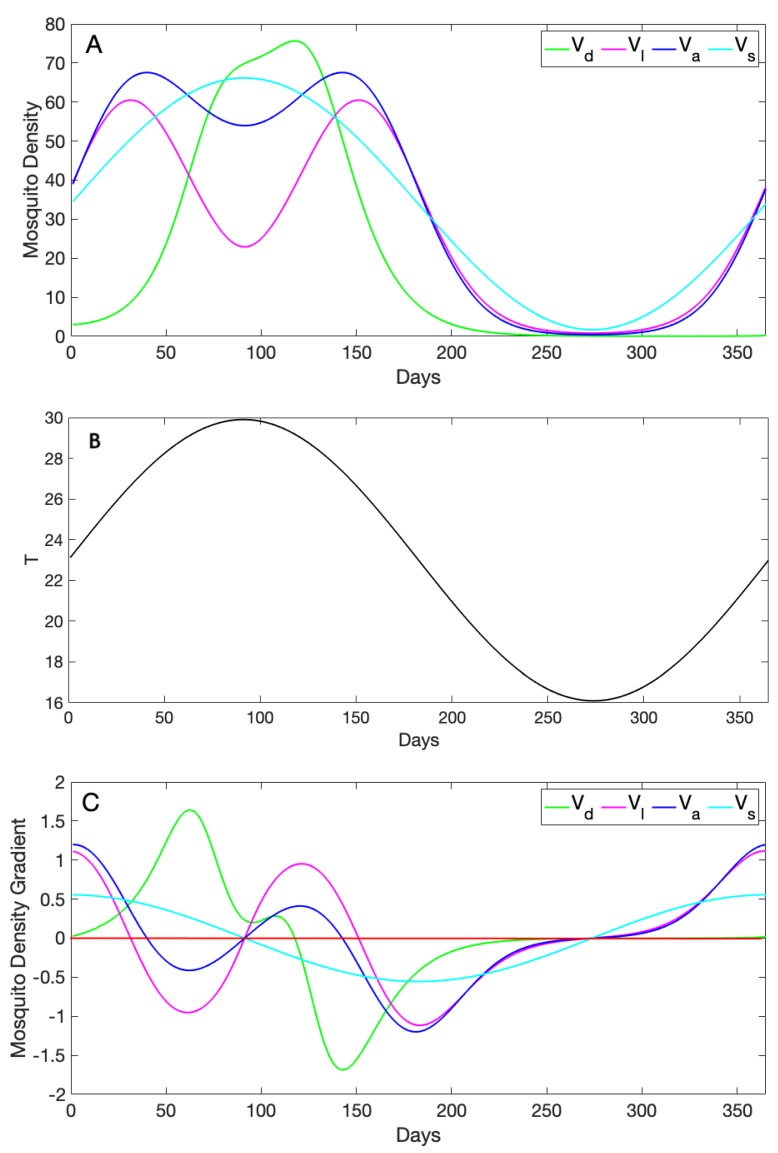
(**A**) Daily adult female mosquito densities approximated using four forms, namely the sine wave $V_{s}$ with initial $V_{0}=33$ and amplitude of 0.45, the adult trait-dependent form $V_{a}$, the life stage trait-dependent $V_{l}$, and the dynamical density $V_{d}$; the parameter values used to solve the ode model are given in Table 1. (**B**) Daily temperature given by a sine function ($T_{0}(1+B\sin(2\pi t/365$ with $T_{0}=23$ and B=0.3) evaluated for 365 days used to evaluate our thermal traits incorporated into the dynamical model. (**C**) Densities’ first derivatives calculated to show how each curve changes its gradient as it varies with temperature. The red line shows when the gradient is zero; positive gradient values (above the red line) mean that the density increases; and negative values of the gradient (below the red line) mean that the curve decreases.

The first regime (Figure 4) followed a sine wave that started at an initial temperature of $T_{0}=23$ °C and ranged from a minimum of 16 °C to a maximum of 30 °C (i.e., this was the same forcing used in the previous section). In this case, the predictions of the four models were largely very different in both pattern and value. However, $V_{l}$ and $V_{a}$ agreed when the temperature was below 25 °C. This is because of the similarities in their formula and the way they incorporate thermal traits. As temperature increased, $V_{d}$ increased exponentially, while the rest of the curves had a more linear increase at first. We note that the peak of $V_{d}$ was delayed due to incorporating the dynamics for each stage explicitly in the model, whereas the rest of the curves were evaluated directly at each temperature. All three densities that included trait information ($V_{d},\;V_{l},\;V_{a}$) decreased as temperatures peaked or approached a minimum, which reflected the high mortality of vectors at very high and very low temperatures. $V_{s}$ decreased as well, but not as drastically, since it followed the temperature sine wave patterns. Further, $V_{s}$ neither decreased nor slowed during high temperature periods, a qualitatively different density prediction compared to the other three models in this scenario.

We can additionally explore the change in the predicted densities across time by comparing their gradients (i.e., the first derivative of the curve, so that a positive value indicates that the density increased, while a negative value indicated a decrease; see Figure 4C). Local extrema (minima or maxima) occurred when the gradient reached zero. In Figure 4C, we can see that three of the models reached extrema when temperature peaked (∼Day 90); however, at that point, the sine wave was a maximum, but the other two were at a minimum. Those two models had two maxima at more intermediate temperatures, although they did not reach those maxima at the same time. In contrast, although the dynamical model also exhibited only a single extrema, a maximum, it was later that the sine wave and after the peak temperature, but before, the other two models reached their second maxima.

**Figure 5:**
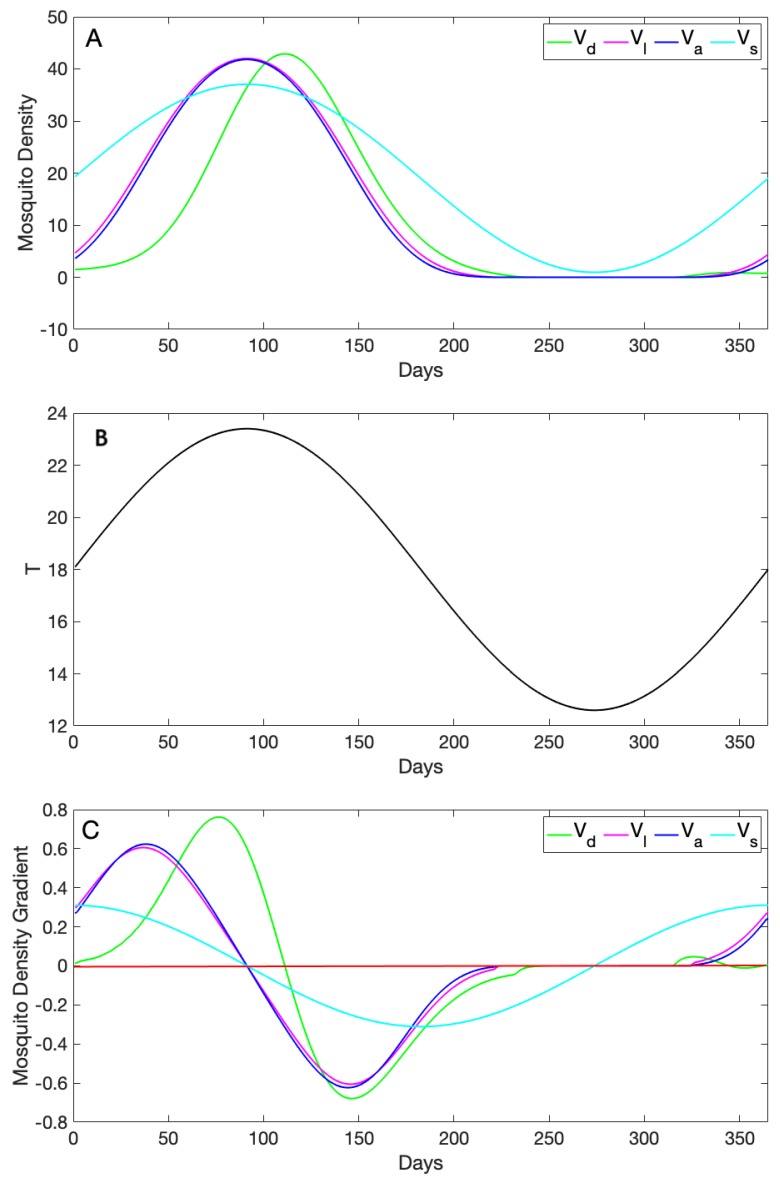
(**A**) Daily adult female mosquito densities approximated using four forms, namely the sine wave $V_{s}$ with initial $V_{0}=19$ and amplitude of 0.95, the adult trait-dependent form $V_{a}$, the life stage trait-dependent $V_{l}$, and the dynamical density $V_{d}$; the parameter values used to solve the ode model are given in Table 1. (**B**) Daily temperature given by a sine function ($T_{0}(1+B\sin\left(2\pi t/365\right)$ with $T_{0}=18$ and B=0.3) evaluated for 365 days used to evaluate our thermal traits incorporated into the dynamical model. (**C**) Densities’ first derivatives calculated to show how each curve changes its gradient as it varies with temperature. The red line shows when the gradient is zero; positive gradient values (above the red line) mean that the density increases; and negative values of the gradient (below the red line) mean that the curve decreases.

In contrast to the first case, the dynamics of all of the models were much more similar when temperature varied around a lower temperature, specifically when the starting temperature was 18 °C with a range of 12–23 °C (Figure 5). This low range led all density forms to have global optimums compared to local optimums in the previous regime. This was because, in this thermal regime, the temperature was fluctuating along the linear part of all (or nearly all) of the thermal trait curves (see Appendix A for the plots of all thermal traits from 0 °C to 50 °C). In particular, here, the two forms $V_{a}$ and $V_{l}$ were very similar, since they combined traits together in similar ways. $V_{s}$ had a sine shape with a lower peak compared to $V_{a}$ and $V_{l}$. In contrast, there was a 20-day lag between when $V_{d}$ was observed to peak compared to the peaks in the other densities (Figure 5C). This is because in the dynamical model, the impact of temperature must propagate through the life stages, instead of appearing immediately. This was also why in the first regime we explored, we did not see the extreme peaks and valleys due to going “over the hump” of the thermal curves.

### 3.3. Comparison to Observational Abundance Data on A. aegypti

We further wished to explore the extent to which any of the density forms presented were consistent with observational data on the abundances of *A. aegypti*. We compared model predictions to data collected as part of a long-term program of entomological surveillance (“Intelligent Dengue Monitoring System”) in the city of Vitòria in Brazil Lana et al. [15]. *A. aegypti* mosquitoes were collected weekly from mosquito traps placed in 80 neighborhoods of the city across four years (January 2008 to December 2011), resulting in 208 weeks of *A. aegypti* mosquito abundance. Concurrent weekly temperature data at the city level were obtained from the International Research Institute for Climate and Society (IRI) platform at Columbia University Land Institute.

**Figure 6:**
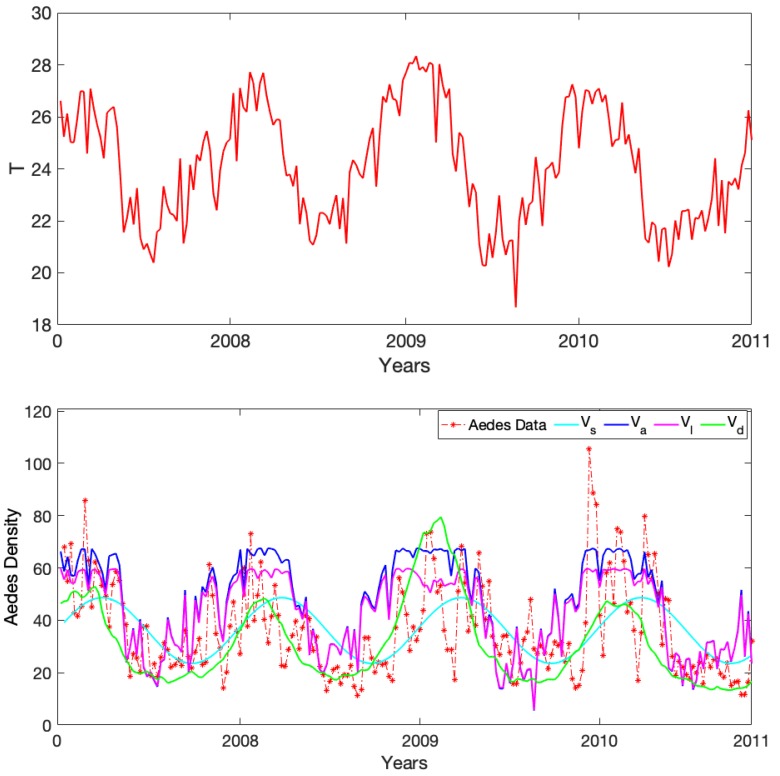
(**Top**) Vitòria city’s weekly average temperature during the period of the study. (**Bottom**) Vitòria city’s *Aedes* weekly abundance data in red stars and our density estimates evaluated at the weekly temperature in Vitòria city, Brazil. The sine wave density $V_{s}$ in cyan blue, the adult trait-dependent density $V_{a}$ in dark blue, the life stage trait-dependent density $V_{l}$ in magenta, and the dynamical density $V_{d}$ in green. Mosquito and temperature data both from [15].

We used weekly average temperatures from Vitòria as inputs for the three trait-dependent models (i.e., to evaluate *A. aegypti* mosquito traits each week) in order to obtain the weekly prediction of each model. Because of the delayed response shown in $V_{d}$ dynamics, we defined a burning time of eight weeks to reduce the lag between $V_{d}$ patterns and *A. aegypti* abundance. In addition, we adjusted the temperature-independent model parameters *k* and $k_{j}$ to make $V_{d}$ close to *A. aegypti* abundance in amplitude. We did not tune any of the parameters in $V_{l}$ and $V_{a}$ since they were all temperature-dependent. For the sine wave density, $V_{s}$, we changed the frequency from daily to weekly and fit the amplitude and intercept using least squares (i.e., as a simple linear regression model). The fitted version equation is given by $V_{s}=36.31+0.35sin\left(2\pi t/52\right)$. In Figure 6 Top, we show the weekly temperature data for Vitòria city, and in Figure 6 Bottom, we show the corresponding predictions for our four models together with the observed *Aedes* abundance data.

To investigate how close each model was to the observed data, we calculated the mean squared error (MSE) between the prediction of each model and the data (Table 2). The smaller the MSE, the closer the prediction to the data on average. Not surprisingly, the closest approximation was $V_{s}$, which was the only model that was fitted to the abundance data directly. By this metric, the second best was $V_{l}$ (the life stage trait-dependent curve), followed by the dynamical curve $V_{d}$ (that also incorporated life stage traits). The adult trait-dependent density model $V_{a}$ was the poorest fitting model, indicating that incorporating juvenile traits may improve predictions of adult dynamics.

## 4. Discussion

Vector density is an essential component of vector-borne disease transmission as it partially determines interaction rates between vectors and hosts. For instance, a location with a high vector density increases the chance for contact with the host, which increases disease risk and facilitates disease spread [26,27]. However, measuring mosquito abundance in the field can be challenging, and the role climate factors play in the global distribution and dynamics of mosquitoes including *A. aegypti* remains unclear [28,29]. Instead of relying exclusively on measurements, mathematical models could potentially help bridge the gap between what we know about vector biology and the dynamics of vector populations. The dynamics of vector populations in general, including mosquito populations, are driven by the dynamics of all life stages and by the traits of the vectors across stages. Thus, better understanding mosquito traits and how they determine dynamics have knock-on impacts for understanding constraints on transmission of VBDs like dengue and for planning and implementing intervention strategies.

In this study, we compared four models of differing complexity that could be used to approximate vector density for *A. aegypti* based on temperature. The model formulations we explored ranged along a spectrum based on the amount of detail they included on mosquito thermal traits from none (just a simple sine wave to represent “seasonality”) to a dynamical model including separate equations for each of the four mosquito life history stages. When each model was allowed to vary as it was forced by extrinsic seasonal temperature forcing, the models exhibited patterns that differed both quantitatively and qualitatively. For example, in the lower temperature regime, the qualitative patterns were fairly similar across models (primarily differing in the exact values observed; Figure 5), while in the higher temperature regime, both significant qualitative patterns (e.g., dual peaks in some models versus single peaks in others), as well as differences in quantitative predictions (e.g., heights of peaks, Figure 4) existed. Unlike the rest of the density forms, the simple sine wave cannot explicitly capture the effects of the unimodality of thermal traits on mosquito density.

Ideally, the models constructed should be able to serve dual purposes. That is, they should be useful for testing our understanding, as well as for making predictions. To test the usefulness of the models for this second purpose, we explored the extent to which each of the models, with some tuning, could be used to predict observational data on *A. aegypti* abundance data collected in Vitòria city, Brazil, based on weekly average temperatures. We explicitly fit the simple sine wave function to the data. This served as a kind of null model; this is perhaps the most efficient simple model we might expect to capture the gross patterns in these data. The other models were tuned to get them as close as possible to the observed data without changing the temperature-dependent relationships encoded in the models (i.e., only the non-temperature-dependent parameters were adjusted). Not surprisingly, the sine wave function, the only explicitly fit to the observed data, gave the smallest error. However, the other models also were able to capture many of the features of the data; for example, capturing approximate timing of peaks and troughs in abundances.

Each of the models with different amounts of detail are likely to be useful in different situations. For example, if we are primarily interested in approximating the number of mosquitoes over time for a given location based on historical data, then using a simple sine wave might be sufficient. However, the fit of that model is not likely to be useful to predict dynamics in another setting, for example in a city with a very different climate. In contrast, the models that are built from experimental data are quite general because they include biological information about mosquito traits. With potential daily temperature patterns, we could make predictions about how the mosquito dynamics would look if *A. aegypti* invades a new location based on any of the trait based models. Indeed, the intermediate complexity model versions compared here ($V_{a}$ and $V_{l}$) were successfully used to quantify how the basic reproductive ratio of *falciparum* malaria depends on temperature, and the predictions were congruent with the observed entomological inoculation rate [9]. Thus, for capturing large scale patterns or extrapolating to new locations, any of the models that included traits could potentially be useful. We expect that our most detailed model that explicitly included the dynamics of juvenile stages would be most useful when data on larval mosquitoes are collected instead of on adults or when the effectiveness of interventions to reduce larval populations is being explored. Models that do not include details on juveniles cannot be used for that purpose. Thus, the choice of which model to use must be guided based on modeling goals and how this matches with modeling assumptions and with data types and availability [18,19,30,31].

## Figures and Tables

**Table 1:** Mosquito traits used across the three trait-dependent models explored. For each trait, we include the parameter, trait description, unimodal function assumed (for thermal traits), and The value of the trait or values of parameters of the curve. We provide the references and the curves associated with the temperature-dependent traits in Appendix A.

Parameter	Description	Function	Value	Units
lf	Adult mosquito lifespan (μ=1/lf)	quad	$qd=-0.0011,\;T_{m}=36.1065,\;T_{0}=9.1088$	day
$p_{EA}$	Eggs-to-adult survival probability	quad	$qd=-0.0006,\;T_{m}=38.29,\;T_{0}=13.56$	-
*F*	Eggs per female per day	Brière	$\alpha =0.0086,\;T_{m}=34.61,\;T_{0}=14.58$	day^−1^
$\rho_{E}$	Eggs development time	quad	inter=87.1722,slope=1.1575,qd=0.0136	day
$\rho_{L}$	Larval development time	quad	inter=201.2429,slope=14.0345,qd=0.2310	day
$\rho_{P}$	Pupal development time	quad	inter=20.5074,slope=1.0954,qd=0.0153	day
$1/\rho_{A}$	Mosquito development rate	Brière	$\alpha =0.0000786,\;T_{m}=39.17,\;T_{0}=11.36$	day^−1^
$p_{E}$	Eggs survival probability	Brière	$\alpha =0.00077,\;T_{m}=30.33,\;T_{0}=9.395$	-
	$\mu_{E}=\left(1-p_{E}\right)/\rho_{E}$			
$p_{L}$	Larval survival probability	Brière	$\alpha =0.00046,\;T_{m}=36.79,\;T_{0}=2.354$	-
	$\mu_{L}=\left(1-p_{L}\right)/\rho_{L}$			
$p_{P}$	Pupal survival probability	Brière	$\alpha =0.0349,\;T_{m}=37.4679,\;T_{0}=9.96278$	-
	$\mu_{P}=\left(1-p_{P}\right)/\rho_{P}$			
*k*	Temperature-independent mortality rate	-	0.486	day^−1^
$k_{j}$	Density-dependent mortality rate	-	0.09	day^−1^

**Table 2:** Mean squared error (MSE) calculated between *A. aegypti* abundance data and the predictions from our suite of models for the mosquito density $V_{d}$, $V_{l}$, $V_{a}$, and $V_{s}$.

Model	$V_{d}$	$V_{l}$	$V_{a}$	$V_{s}$
MSE	279	255	316	227

**Figure A1:**
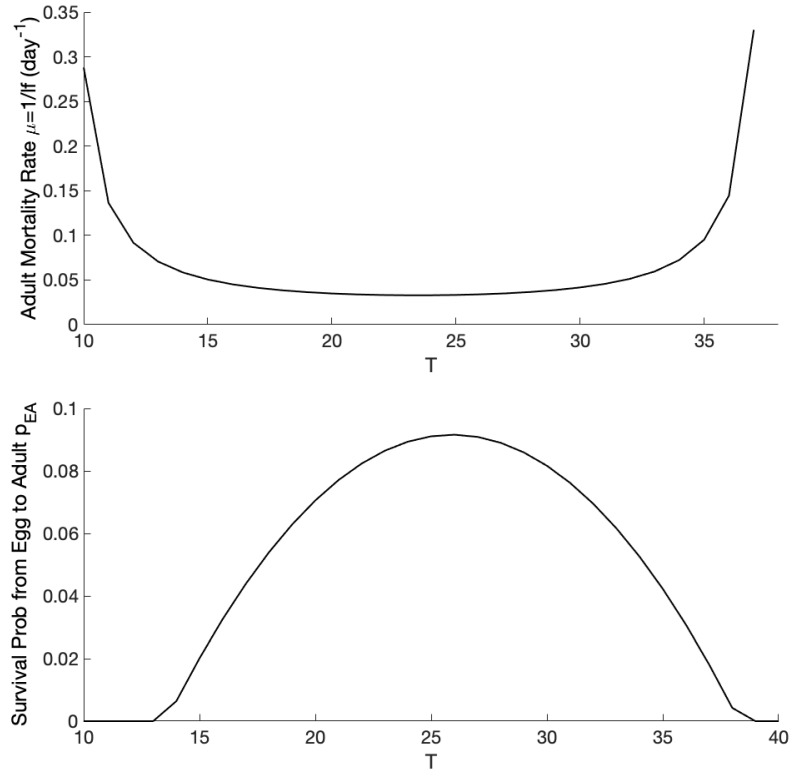
Adult mosquito mortality rate μ and the survival probability from eggs to adult $p_{EA}$ as they vary with the temperature range of 0 °C to 50 °C [24,32].

**Figure A2:**
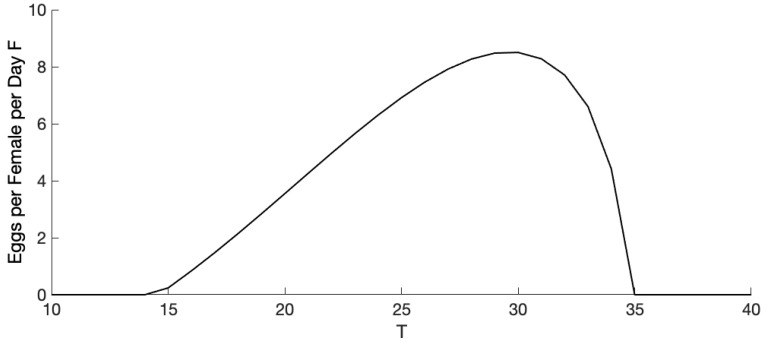

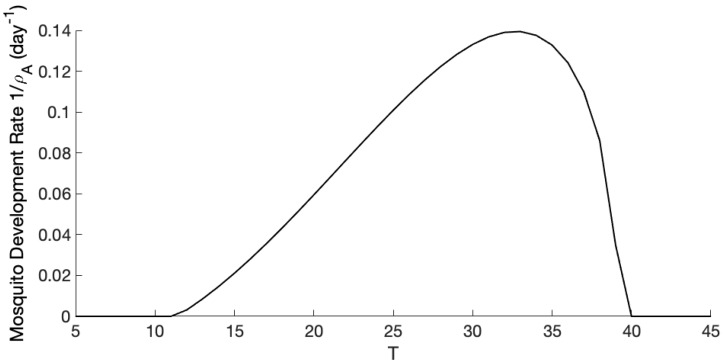
Adult mosquito fecundity *F* [8,33] and the adult mosquito development rate $1/\rho_{A}$ [24,32] as they vary with the temperature range of 0 °C to 50 °C.

**Figure A3:**
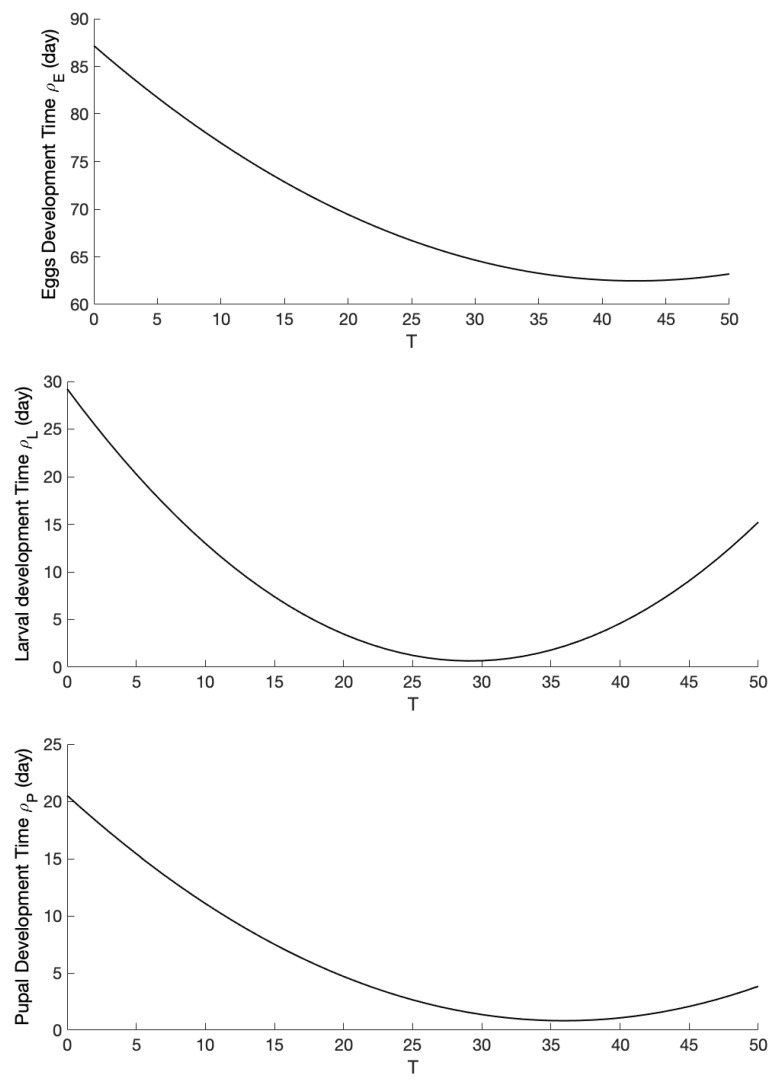
Juvenile stages’ development time, eggs $\rho_{E}$, larvae $\rho_{L}$, and pupae $\rho_{P}$ as they vary with the temperature range of 0 °C to 50 °C [8,33].

**Figure A4:**
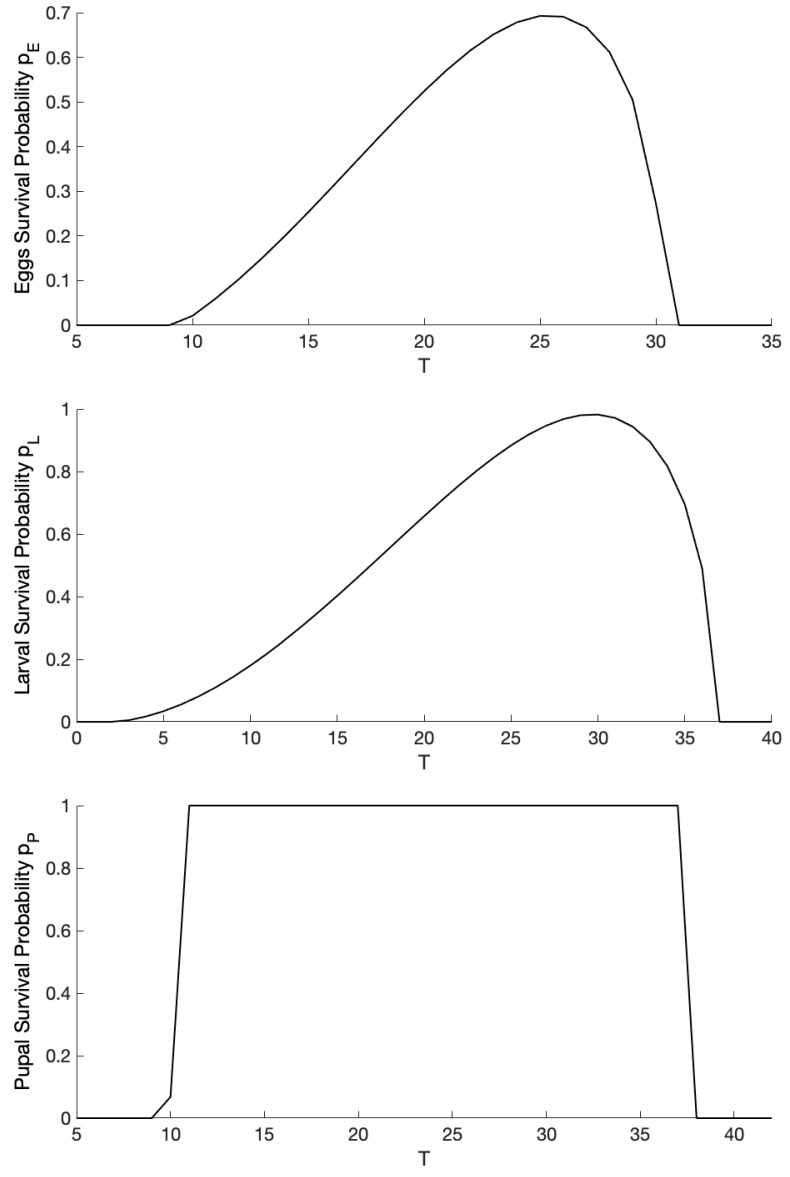
Juvenile stages’ survival probability, eggs $p_{E}$, larvae $p_{L}$, and pupae $p_{P}$ as they vary with the temperature range of 0 °C to 50 °C [34].

**Figure A5:**
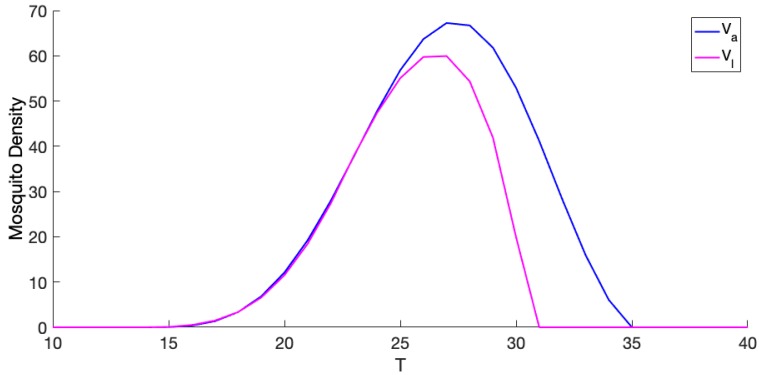
Adult trait-dependent mosquito density $V_{a}$ and life stage trait-dependent mosquito density $V_{l}$ as they vary with the temperature range of 0 °C to 50 °C.

**Figure A6:**
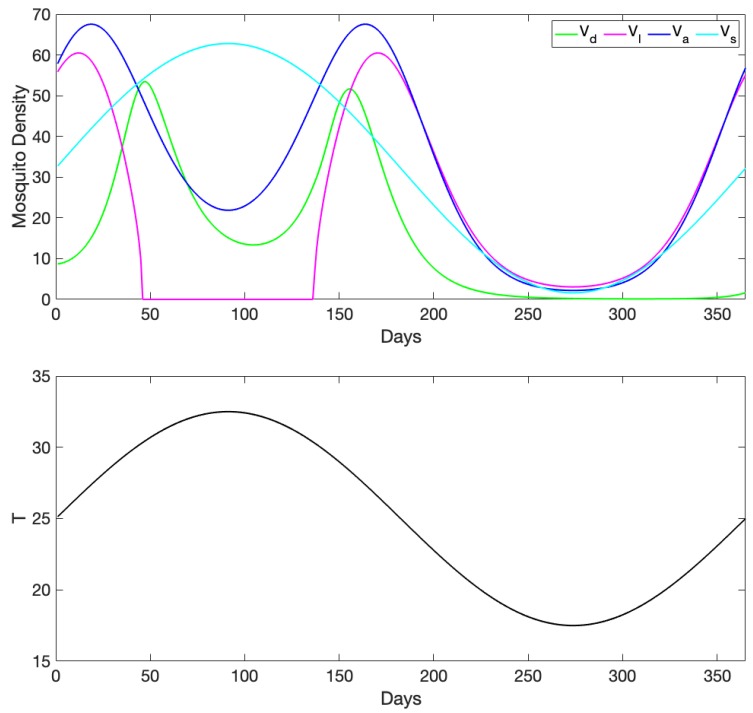

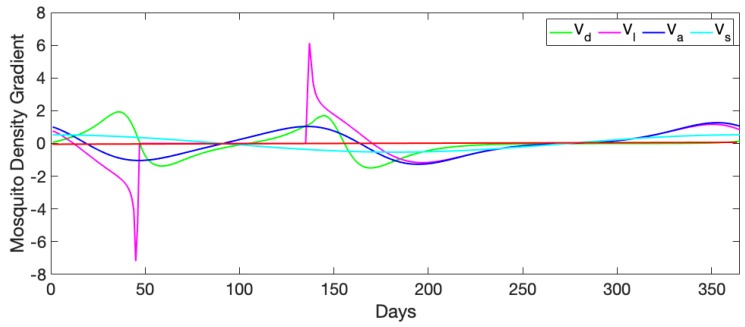
(**Top**) Daily adult females mosquito densities approximated using four forms, namely the sine wave $V_{s}$ with initial $V_{0}=33$ and amplitude of 0.45, the adult trait-dependent form $V_{a}$, the life stage trait-dependent $V_{l}$, and the dynamical density $V_{d}$; the parameter values used to solve the ode model are given in Table 1. (**Middle**) Daily temperature given by a sine function ($T_{0}(1+B\sin(2\pi t/365$ with $T_{0}=25$ and B=0.3) evaluated for 365 days used to evaluate our thermal traits incorporated into the dynamical model. (**Bottom**) Densities’ first derivatives calculated to show how each curve changes its gradient as it varies with temperature. The red line shows when the gradient is zero; positive gradient values (above the red line) mean that the density increases; and negative values of the gradient (below the red line) mean that the curve decreases.

**Figure A7:**
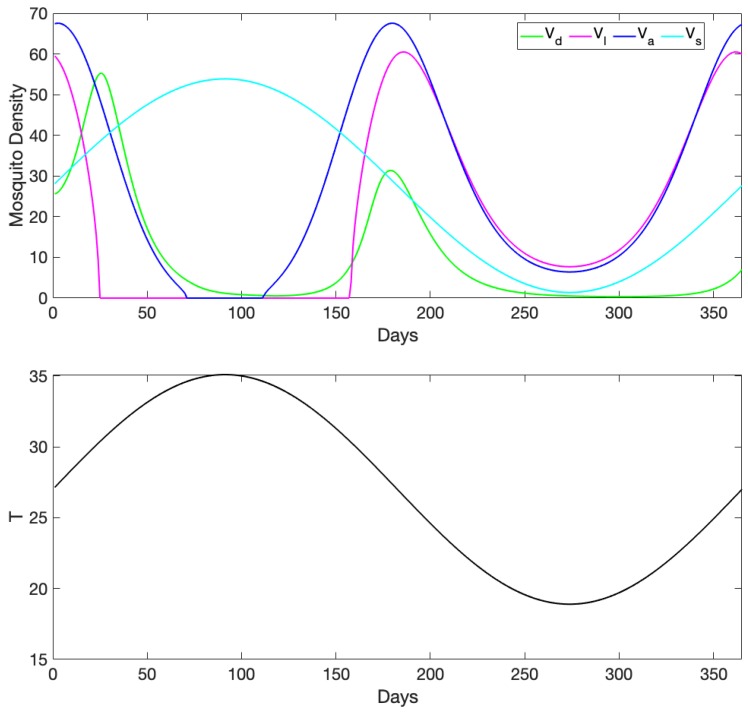

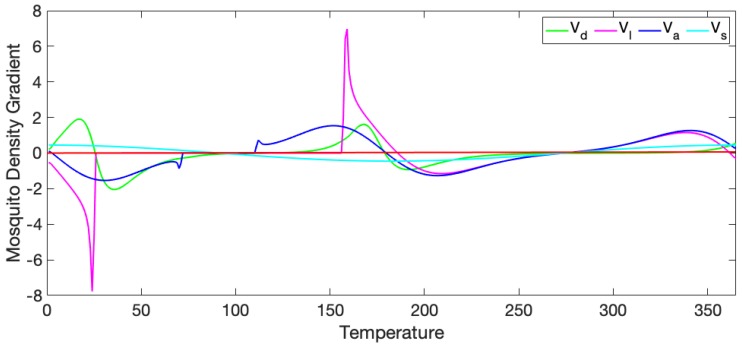
(**Top**) Daily adult females mosquito densities approximated using four forms, namely the sine wave $V_{s}$ with initial $V_{0}=33$ and amplitude of 0.45, the adult trait-dependent form $V_{a}$, the life stage trait-dependent $V_{l}$, and the dynamical density $V_{d}$; the parameter values used to solve the ode model are given in Table 1. (**Middle**) Daily temperature given by a sine function ($T_{0}(1+B\sin(2\pi t/365$ with $T_{0}=27$ and B=0.3) evaluated for 365 days used to evaluate our thermal traits incorporated into the dynamical model. (**Bottom**) Densities’ first derivatives calculated to show how each curve changes its gradient as it varies with temperature. The red line shows when the gradient is zero; positive gradient values (above the red line) mean that the density increases; and negative values of the gradient (below the red line) mean that the curve decreases.

## Appendix A

We evaluated the thermal functions described in Table 1 at a temperature varying from 0 °C to 50 °C to look at *Aedes aegypti* adult and juvenile trait responses to temperature. Then, with these thermal curves, we looked at the total mosquito densities $V_{a}$ and $V_{l}$ variation with temperature.
